# Reversing the Tide: Improving the Recruitment of Patients With Inflammatory Bowel Disease in Clinical Trials in the United States

**DOI:** 10.1093/crocol/otaa021

**Published:** 2020-04-03

**Authors:** Parakkal Deepak

**Affiliations:** Division of Gastroenterology and Inflammatory Bowel Diseases Center, Washington University in Saint Louis, St. Louis, Missouri, USA

**Keywords:** inflammatory bowel disease, Crohn’s disease, ulcerative colitis, clinical trials, randomized controlled trials, RCT, eligibility, trends, characteristics, enrollment, participation

## Abstract

While the number of inflammatory bowel disease (IBD) clinical trials has been increasing, there is decreasing participation in these trials with under-representation of younger patients and those with milder disease and in community settings. Innovative methods to increase recruitment and participation through the use of social media tools, apps, electronic medical record-based patient finding algorithms, and remote monitoring methods are suggested.

Crohn’s disease (CD) and ulcerative colitis (UC) are inflammatory bowel diseases (IBDs) that have emerged as global diseases that affect over 2 million individuals in North America, 3.2 million in Europe, and many millions more worldwide.^1^ While several medications across various therapeutic mechanisms are currently available in clinical practice, these are often limited in their effectiveness in achieving and sustaining remission over the lifetime of the individual and/or limited by the eventual development of side-effects. The limitations of the current medications are highlighted by the almost 1500 clinical trials including randomized controlled trials (RCTs) listed on ClinicalTrials.gov, that are available to patients with IBD in the United States.^2^ However, there has been increasing recognition of issues affecting patient recruitment into these trials that prolongs the time needed to move a promising mechanism from bench to the bedside as well affect the generalizability of the trial results to clinical practice settings.

In this issue, Johnson et al^3^ performed a cross-sectional study within the IBD Partners cohort comparing patients with current or prior participation in an RCT of a medical therapy for IBD to those without any RCT participation. They also examined trends in participation in the cohort during the calendar years 2011–2018 compared to the listing of IBD clinical trials on Clinicaltrials.gov. They demonstrated a decline in RCT participation within IBD Partners cohort with an underrepresentation of younger patients, patients followed in community settings, and patients with more mild disease in RCTs for IBD. Furthermore, despite non-participation in RCTs, the non-RCT group had mean disease activity scores that did not meet remission thresholds, suggesting a need for better disease control potentially with alternate therapies that may only be accessible through clinical trials.

Prior publications involving the Crohn’s and Colitis Foundation have highlighted, through patient focus groups, various methods to improve participation in clinical trials including better communication to prospective patients, reduced length of trial and time commitment, lowering placebo rates, the option of open-label extension, and importantly, the support of the patient’s primary IBD provider.^2^ A more quantitative approach to better understand patient preferences for clinical trial design attributes was carried out in 949 adult IBD patients recently by Wood et al^4^ using a choice-based conjoint survey. This study modeled significant increases in recruitment with lower placebo rates, reduced number of endoscopies, less time commitment, open-label extension, and increased involvement of participant’s primary GI physician. The strongest effect, however, was found with increased monetary compensation for participation in the clinical trial. They additionally found that younger patients would potentially fall under the “persuadable” group for participation in clinical trials.

The findings of Johnson et al^3^ suggest an urgent need for improvement in trial design, as highlighted by the decline in participation within the IBD Partners cohort. This is perhaps even more concerning because this cohort likely represents patients who are even more motivated than the usual clinic patient to participate in research that advances the care of patients with IBD. This may also highlight the need for novel ways to recruit patients using social media tools going beyond traditional methods such as word-of-mouth, radio, and television advertisements. Apps offer another potentially powerful way to connect with prospective research participants since the majority of Americans go to the internet first for health information.^5^ Apps such as Novartis’s Clinical Trial Seek attempt to make the information on cancer clinical trials more easily accessible to the lay person by aggregating information from ClinicalTrials.gov and presenting it via a user-friendly interface.^5^ The Clinical Trial Finder on the Crohn’s and Colitis Foundation website similarly aggregates information on IBD clinical trials listed on ClinicalTrials.gov and allows for narrowing the search using variables such as zip code, gender, type of IBD, distance to trial site, phase, and type of trial.

Another under-represented group appears to be patients in a community setting. Many of the newer clinical trials are increasingly attempting to directly engage with community sites for patient recruitment. However, successful participation of community centers also requires a reasonably large cohort of IBD patients given the many criteria as well as an investment of resources both in research coordinators and for expertise in regulatory oversight, budgeting, and contracts. Under-representation of patients from the community setting may also be partly the result of a lack of information about potential clinical trials, both on the part of the provider as well as the patient. Many larger health-care systems have community sites working in a hub and spoke model where imaginative solutions spanning the healthcare system such as patient identification algorithms implemented on a common electronic medical record system followed by inbox messages to the treating clinician and apps specific to the health system such as Stanford’s SCI Cancer Clinical Trials and Thomas Jefferson University’s Clinical Trials—TJU apps, may help to increase patient recruitment from community sites.^5^

Finally, additional barriers such as the need to travel distances from the community to the tertiary-IBD center may be addressed through the use of remote monitoring of patient-reported symptoms and any safety concerns through apps and/or telemedicine, remote collection of blood/stool-based biomarkers and in-person visits during follow-up limited to the need for objective assessment of mucosal inflammation. The Food and Drug Administration has launched a MyStudies app which is secure and supports auditing necessary for compliance with 21 Code of Federal Regulations Part 11 and the Federal Information Security Management Act, configurable for different therapeutic areas, and health outcomes, and the data storage environment is partitioned to support multi-site trials or “distributed database” studies.^6^ It is also open-source with 2 versions of the app, including one built for Apple’s ResearchKit framework, and another built on the open-source ResearchStack framework, which runs on Google’s Android. Such initiatives will allow remote data collection and management of clinical trials to further improve patient recruitment from sites that are far away from the tertiary IBD centers.

## Notes

Supported by: P.D. is supported by a Junior Faculty Development Award from the American College of Gastroenterology.
